# Emergence of HIV-1 drug resistance mutations among antiretroviral-naïve HIV-1-infected patients after rapid scaling up of antiretroviral therapy in Thailand

**DOI:** 10.1186/1758-2652-15-12

**Published:** 2012-03-12

**Authors:** Somnuek Sungkanuparph, Chonlaphat Sukasem, Sasisopin Kiertiburanakul, Ekawat Pasomsub, Wasun Chantratita

**Affiliations:** 1 Division of Infectious Diseases, Department of Medicine, Faculty of Medicine Ramathibodi Hospital, Mahidol University, Bangkok, Thailand; 2 Virology and Molecular Microbiology Unit, Department of Pathology, Faculty of Medicine Ramathibodi Hospital, Mahidol University, Bangkok, Thailand

## Abstract

**Background:**

After rapid scaling up of antiretroviral therapy in HIV-1-infected patients, the data of primary HIV-1 drug resistance in Thailand is still limited. This study aims to determine the prevalence and associated factors of primary HIV-1 drug resistance in Thailand.

**Methods:**

A prospective observational study was conducted among antiretroviral-naïve HIV-1-infected Thai patients from 2007 to 2010. HIV-1 subtypes and mutations were assayed by sequencing a region of HIV-1 pol gene. Surveillance drug resistance mutations recommended by the World Health Organization for surveillance of transmitted HIV-1 drug resistance in 2009 were used in all analyses. Primary HIV-1 drug resistance was defined as the presence of one or more surveillance drug resistance mutations.

**Results:**

Of 466 patients with a mean age of 38.8 years, 58.6% were males. Risks of HIV-1 infection included heterosexual (77.7%), homosexual (16.7%), and intravenous drug use (5.6%). Median (IQR) CD4 cell count and HIV-1 RNA were 176 (42-317) cells/mm^3 ^and 68,600 (19,515-220,330) copies/mL, respectively. HIV-1 subtypes were CRF01_AE (86.9%), B (8.6) and other recombinants (4.5%). The prevalence of primary HIV-1 drug resistance was 4.9%; most of these (73.9%) had surveillance drug resistance mutations to only one class of antiretroviral drugs. The prevalence of patients with NRTI, NNRTI, and PI surveillance drug resistance mutations was 1.9%, 2.8% and 1.7%, respectively. From logistic regression analysis, there was no factor significantly associated with primary HIV-1 drug resistance. There was a trend toward higher prevalence in females [odds ratio 2.18; 95% confidence interval 0.896-5.304; p = 0.086].

**Conclusions:**

There is a significant emergence of primary HIV-1 drug resistance in Thailand after rapid scaling up of antiretroviral therapy. Although HIV-1 genotyping prior to antiretroviral therapy initiation is not routinely recommended in Thailand, our results raise concerns about the risk of early treatment failure in patients with primary HIV-1 drug resistance. Interventions to prevent the transmission of HIV-1 drug resistance and continuation of surveillance for primary HIV-1 drug resistance in Thailand are indicated.

## Background

In Thailand, the disease burden from HIV/AIDS resulting from the epidemic in the 1990s remains high [1]. Although the incidence rate of HIV-1 infection in Thailand from 2001 to 2009 has decreased by more than 25% [2], the accumulated number of HIV-1-infected persons is still high. Currently, an estimated 530,000 people are living with HIV in Thailand [2]. Combination antiretroviral therapy (ART) has significantly reduced mortality and morbidity since its introduction in Thailand [3-5]. Since 2001, the government has committed to providing ART free of charge to people living with HIV under the National Access to Antiretroviral Program for People Living with HIV/AIDS (NAPHA) [6]. The subsequent production and use of generic drugs led to more than an eight-fold expansion in treatment provision between 2001 and 2003 [7]. Since 2006, with rapid growth of NAPHA, it has been transformed into the National AIDS Program under the management of the National Health Security Office. According to the UNAIDS 2010 report, 216,118 persons were receiving ART in December 2009, and the number of life years among adults gained due to ART between 1996 and 2009 is 389,000 [2].

Despite these successes, HIV-1 drug resistance (HIVDR) is a major reason for treatment failure during rapid scaling up of ART in Thailand [8,9]. According to the Thai national treatment guidelines, non-nucleoside reverse transcriptase inhibitor (NNRTI)-based regimens are recommended as first-line regimens [10]. Approximately 5% to 10% of patients receiving ART have experienced treatment failure and HIVDR [10]. Previous survey studies in Thailand had shown low prevalence of transmitted HIVDR among Thai patients with early HIV-1 infection [11,12]. Recently, a study in Thailand demonstrated the transmission of HIVDR in antiretroviral-naïve HIV-1-infected patients [13]. This threatens the effectiveness of rapidly scaled up first-line ART in the country.

Primary HIVDR means that there is increased resistance of HIV-1 to antiretroviral drugs seen in individuals who have never received ART and presumably have been infected with a drug-resistant virus [14]. The prevalence of primary HIVDR has been well reported in the United States and Europe, and ranges from 6.2% to 21% [15-18]. A study in Asia has recently reported the prevalence of primary HIVDR at 13.8% [19]. In resource-limited settings where ART is being scaled up, the World Health Organization (WHO) recommends the surveillance of primary HIVDR [20]. To date, after a decade of ART scaling up, there is limited published information regarding primary HIVDR in Thailand. This study was aimed at determining the prevalence of HIVDR and associated factors among antiretroviral-naïve patients in Thailand.

## Methods

A cross-sectional study was conducted among antiretroviral-naïve HIV-1-infected patients who recently visited the Infectious Disease Clinic at Ramathibodi Hospital, a university hospital, between January 2007 and December 2010. This clinic primarily serves patients from Bangkok and its peripheral areas. Patients with a history of any exposure to antiretroviral drugs, including mono or dual therapy, or prevention of mother to child transmission, were excluded. Ethics approvals were obtained from local institutional review boards. Informed consent was obtained prior to genotypic resistance testing.

All plasma samples, HIV-1 pol nucleotide sequencing of reverse transcriptase and protease region was carried out using TRUGENE HIV-1 Genotypic Assay in conjunction with the Open Gene automated DNA sequencing system (Visible Genetics, Toronto, Canada). Testing involved simultaneous clip sequencing of protease and codons 35-244 of the RT from the amplified cDNA in both the 3' and 5' directions. Sequences were aligned and compared with a lymphoadenopathy-associated virus type 1 (HIV-B-LAV1) consensus sequence using Visible Genetics Gene Librarian software [21,22]. Genotypic HIV-1drug resistance testing was performed with externally quality controlled. Subtype was determined on the basis of genotyping of pol gene. Surveillance drug resistance mutations (SDRMs) recommended by WHO for surveillance of transmitted HIVDR in 2009 [23] were used in all analyses. HIVDR in a patient was defined as the presence of at least one SDRM.

Mean (± standard deviation, SD), median (interquartile range, IQR) and frequencies (%) were used to describe patients' characteristics. Categorical variables between the two groups were compared using Chi square or Fisher's exact test as appropriate. Continuous variables between the two groups were compared using Student's t test and Mann-Whitney U test as appropriate. Logistic regression analysis was used to determine factors associated with HIVDR. A p value of < 0.05 was considered to be statistically significant. All analyses were performed using SPSS version 16.0 (SPSS Inc., Chicago, Illinois, USA).

## Results

A total of 466 patients were included in this analysis. The mean (SD) age was 38.8 (11.4) years. In total, 263 (58.6%) patients were males. Risks of HIV-1 infection were heterosexual (77.7%), homosexual (16.7%), and intravenous drug use (5.6%). Forty-six (9.9%) and 32 (6.9%) patients had co-infection of hepatitis B virus and hepatitis C virus, respectively. Median (IQR) CD4 cell count and HIV-1 RNA were 176 (42-317) cells/mm^3 ^and 68,600 (19,515-220,330) copies/mL, respectively. Of 466 patients, 405 (86.9%) were infected with HIV-1 subtype CRF01_AE. Subtype B was found in 40 (8.6) patients. Other subtypes (4.5%) were CRF07_BC, CRF03_AB, CRF02_AG, CRF12_BF, D and K.

The prevalence of primary HIVDR was 4.9%. The prevalence of patients with nucleoside reverse transcriptase inhibitor (NRTI), NNRTI and protease inhibitor (PI) SDRMs was 1.9%, 2.8% and 1.7%, respectively. Seventeen (3.8%) patients had at least one SDRM to only one class of antiretroviral drugs. Five (1.1%) patients had both NRTI and NNRTI SDRMs. Only one patient had SDRMs to three classes of antiretroviral drugs. Table 1 shows SDRMs observed in 23 patients with HIVDR. The comparison of characteristics between patients with and without HIVDR is summarized in Table 2. From logistic regression analysis, there was no factor significantly associated with HIVDR. There was a trend toward higher prevalence in females [OR = 2.18; 95% CI 0.896-5.304; p = 0.086].

**Table 1:** Distribution of SDRMs in 23 patients with primary HIVDR*

SDRMs	Number of patients	Prevalence (%)
NRTI-SDRMs	9	1.9

M41L	3	0.6

K65R	1	0.2

D67N	1	0.2

T69D	1	0.2

V75M	1	0.2

M184V	3	0.6

M184I	1	0.2

L210W	1	0.2

T215Y	1	0.2

T215S	1	0.2

K219Q	1	0.2

K219R	1	0.2

NNRTI-SDRMs	13	2.8

K101E	1	0.2

K103N	3	0.6

K103S	1	0.2

V106A	1	0.2

V106M	1	0.2

Y181C	4	0.9

Y181I	1	0.2

Y188L	1	0.2

G190S	1	0.2

PI-SDRMs	8	1.7

M46I	1	0.2

M46L	1	0.2

I47V	1	0.2

G48M	1	0.2

I54L	1	0.2

I54T	1	0.2

I84A	1	0.2

L90M	6	1.3

*some patients had more than one SDRM

**Table 2:** Comparison of characteristics between patients with and without primary HIVDR

Characteristics	Primary HIVDR		*P *value
		
	Yes *(n = 23)*	No *(n = 443)*	
Age, years, mean ± SD	37.3 (7.9)	38.8 (11.5)	0.517

Male gender, number (%)	9 (39.1)	264 (59.6)	0.080

Risk of HIV-1 infection, number (%)			0.489

Heterosexual	19 (82.6)	343 (77.4)	

Homosexual	2 (8.7)	76 (17.2)	

IVDU	2 (8.7)	24 (5.4)	

HBV co-infection, number (%)	2 (8.7)	44 (9.9)	0.579

HCV co-infection, number (%)	2 (8.7)	30 (6.8)	0.326

CD4, cells/mm^3^, median (IQR)	197 (35-307)	173 (43-318)	0.784

HIV-1 RNA, log copies/mL, median (IQR)	29,600 (3,580-214,000)	70,150 (20,490-220,740)	0.271

HIV-1 subtypes, number (%)			0.551

CRF01_AE	21 (91.4)	384 (86.7)	

B	1 (4.3)	39 (8.8)	

Others*	1 (4.3)	20 (4.5)	

IVDU=intravenous drug use

*including CRF07_BC, CRF03_AB, CRF02_AG, CRF12_BF, D, and K

## Discussion

Primary HIVDR represents a challenge for the treatment of HIV-1 infection because it can reduce the efficacy of first-line ART and may impact clinical outcomes. Emergence of primary HIVDR in resource-limited settings is a concerning consequence of global scaling up of ART. It is established that primary HIVDR will emerge in the region where ART has been widely available for years [20]. After a decade of rapid scaling up of ART in Thailand, primary HIVDR is inevitably anticipated.

The results from the present study have demonstrated that there is an emergence of primary HIVDR in Thailand. The prevalence of primary HIVDR in the present study is 4.9%, approaching WHO's first threshold (5%) of transmitted HIVDR. Previous studies had predicted that transmitted HIVDR would reach 5% after approximately 10 years of ART scaling up [20,24]. Although the term, "transmitted HIVDR", is generally applied only to HIVDR detected in recently infected individuals, the prevalence of primary HIVDR among patients with chronic HIV-1 infection may be even underestimated. Thus, the results from the present study provide data about the likely efficacy of first-line ART in Thailand. For example, the prevalence of primary HIVDR at 4.9% indicates that about 5% of patients initiating first-line ART in Thailand may have early treatment failure. NNRTI-based regimens, which are preferred regimens for first-line ART in Thailand, generally have low genetic barriers for development of resistance, and early treatment failure is likely if the regimen does not consist of three fully active drugs [25,26].

NNRTI SDRMs, M184V and thymidine analogue-associated mutations (TAMs) are the most common SDRMs observed in the present study. These mutations are commonly found in patients failing NNRTI-based regimens, such as zidovudine/lamivudine/nevirapine and zidovudine/lamivudine/efavirenz, which are widely used as first-line ART in Thailand [9]. Recently, various multicentre cohort studies have demonstrated that primary HIVDR is associated with poor treatment outcomes and/or clinical complication [27-29]. They all support the use of genotypic resistance test prior to initiation of ART.

Since 1998, the International AIDS Society-USA Panel had suggested considering resistance testing for antiretroviral-naïve patients in areas with a prevalence of resistance of ≥ 5% [30]. However, a cost-effectiveness study of genotypic resistance testing for antiretroviral-naïve patients with chronic HIV-1 infection has reported that it is cost effective if the prevalence of primary HIVDR is more than 1% [31].

Thailand is an area with predominance of HIV-1 subtype CRF01_AE. Although the prevalence of HIVDR in patients with subtype CRF01_AE is twice that of patients with subtype B in the present study, there was no statistically significant difference. There were also no significant differences in demographic or clinical factors between those with and without primary HIVDR. There was only a trend toward higher prevalence in females from multivariate analysis. Therefore, there is no risk group to consider genotypic testing for primary HIVDR in Thailand. A recent study has shown that ART-naïve patients older than 25 years exhibited significantly higher prevalence of primary HIVDR than younger patients [32]. However, there was no difference of the prevalence of primary HIVDR between these two age groups in the present study.

As ART continues to be scaled up rapidly, it is likely that the prevalence of primary HIVDR continues to increase. It is a national priority to intervene to prevent further transmission of HIVDR. To minimize primary HIVDR in Thailand, strengthening the healthcare system, supporting adherence to therapy, and ensuring a continuous supply of antiretroviral drugs are crucial. At some point, the National AIDS Program in Thailand has to carefully consider the advantages and disadvantages of genotypic testing for primary HIVDR and decide when and how to implement. Future plans have to include strategies to make genotypic testing more accessible with the newer technologies, such as point mutation assays or short sequencing of a specific region of RT gene.

There are some limitations in the present study. Although the patients in this study were those who newly presented to the infectious disease clinic, some patients presented late. They were tested for HIV-1 genotypes at the stage of chronic infection. Some resistance mutations may have reverted to wild type. Thus, the prevalence of primary HIVDR could be underestimated. However, transmitted HIVDR among antiretroviral-naïve patients has been reported to be persistent, ranging from four years to longer than the lifetime of the patient [33]. The prolonged persistence of transmitted HIVDR strongly supports the use of a genotypic resistance test in newly presented patients.

## Conclusions

Primary HIVDR is emerging in Thailand after a decade of rapid scaling up of ART. Although HIV-1 genotyping prior to ART initiation is not routinely recommended in Thailand, continuation of surveillance for primary HIVDR in Thailand is indicated. Public health interventions to prevent the transmission of HIVDR should be implemented on a large scale.

## Competing interests

The authors declare that they have no competing interests.

## Authors' contributions

SS and SK participated in the design of the study, enrolled patients, collected data on patient history, and drafted the manuscript. CS, EP and WC carried out the viral load assays, genotypic drug-resistance test, and subtype analysis. All authors have read and approved the final version of this manuscript.
